# Identification of the functional PD-L1 interface region responsible for PD-1 binding and initiation of PD-1 signaling

**DOI:** 10.1016/j.jbc.2023.105353

**Published:** 2023-10-17

**Authors:** Rachel Carter, Fatimah Alanazi, Amanda Sharp, Jessica Roman, Alessandra Luchini, Lance Liotta, Mikell Paige, Anne M. Brown, Amanda Haymond

**Affiliations:** 1 Center for Applied Proteomics and Molecular Medicine, George Mason University, Fairfax, Virginia, USA; 2 Program in Genetics, Bioinformatics, and Computational Biology, Virginia Tech, Blacksburg, Virginia, USA; 3 Department of Chemistry and Biochemistry, George Mason University, Fairfax, Virginia, USA; 4 Department of Biochemistry, Virginia Tech, Blacksburg, Virginia, USA; 5 Data Services, University Libraries, Virginia Tech, Blacksburg, Virginia, USA

**Keywords:** checkpoint, immunotherapy, inflammation, PD-1 agonist, interfering peptide, T cell activation

## Abstract

The PD-1/PD-L1 checkpoint pathway is important for regulating immune responses and can be targeted by immunomodulatory drugs to treat a variety of immune disorders. However, the precise protein–protein interactions required for the initiation of PD-1/PD-L1 signaling are currently unknown. Previously, we designed a series of first-generation PD-1 targeting peptides based on the native interface region of programmed death ligand 1 (PD-L1) that effectively reduced PD-1/PD-L1 binding. In this work, we further characterized the previously identified lead peptide, MN1.1, to identify key PD-1 binding residues and design an optimized peptide, MN1.4. We show MN1.4 is significantly more stable than MN1.1 in serum and retains the ability to block PD-1/PD-L1 complex formation. We further characterized the immunomodulatory effects of MN1.4 treatment by measuring markers of T cell activation in a co-culture model with ovarian cancer cells and peripheral blood mononuclear cells. We found MN1.4 treatment reduced cytokine secretion and suppressed T cell responses in a similar manner as recombinant PD-L1. Therefore, the PD-L1 interface region used to design MN1.4 appeared sufficient to initiate PD-1 signaling and likely represents the minimum necessary region of PD-L1 required for PD-1 recognition. We propose a peptide agonist for PD-1, such as MN1.4, could have several applications for treating autoimmune disorders caused by PD-1 deficiencies such as type 1 diabetes, inflammatory arthritis, or autoimmune side effects arising from monoclonal antibody-based cancer immunotherapies.

PD-1 (programmed cell death protein 1) is an immune checkpoint receptor that regulates T cell activation and proliferation. When bound by its ligand, programmed death ligand 1 (PD-L1), downstream signaling pathways result in the suppression of T cell responses (1, 2). The PD-1/PD-L1 pathway has primarily been targeted by checkpoint inhibitors in order to activate immune responses against cancer cells, which can upregulate PD-L1. By blocking this interaction, checkpoint inhibitors suppress downstream PD-1 signaling that normally leads to T cell exhaustion (1, 2). Recent studies have shown that mice with PD-1 deficiencies develop several autoimmune diseases, including lupus-like disease, autoimmune dilated cardiomyopathy, and diabetes, suggesting that the PD-1/PD-L1 pathway could also be targeted with agonists for treating autoimmune diseases (3, 4, 5). While the biological function of the PD-1/PD-L1 checkpoint pathway has been well studied, the specific ligand–receptor interactions required for stimulating PD-1 signaling have yet to be fully elucidated. The reported binding affinity for human PD-1/PD-L1 ranges from 1 to 18 μM depending on the expression system (6, 7, 8, 9), and recent studies suggest that agonism vs. antagonism of PD-1 signaling could be modulated based on the binding affinity of the PD-1 targeting molecule. In a recent study, high-affinity mutants of PD-L1 were initially designed to act as PD-1 agonists but inhibited PD-1 signaling when tested *in vitro* (7). Therapeutic anti-PD-1 monoclonal antibodies, designed to block PD-1 signaling, have reported affinities in the low picomolar range (10). While high-affinity therapeutics have been shown to inhibit PD-1 signaling, studies of soluble, cell-free PD-L1 have produced conflicting results. Many studies suggest the presence of soluble PD-L1 in cancer patients correlates to worse clinical outcomes due to activation of PD-1 signaling and subsequent T cell suppression (11, 12, 13). However, a recent study demonstrated that a splice variant of secreted PD-L1 could act as an antagonist for PD-1 (14). To date, most studies with affinity and functional characterization data for PD-1 targeting molecules have been focused on antagonism (15, 16). Therefore, questions remain regarding the modulation of PD-1 agonism vs. antagonism, and further investigation of the precise PD-1/PD-L1 interactions required for downstream PD-1 signaling would provide valuable insights for regulating T cell activation.

## Results

**Figure 1:**
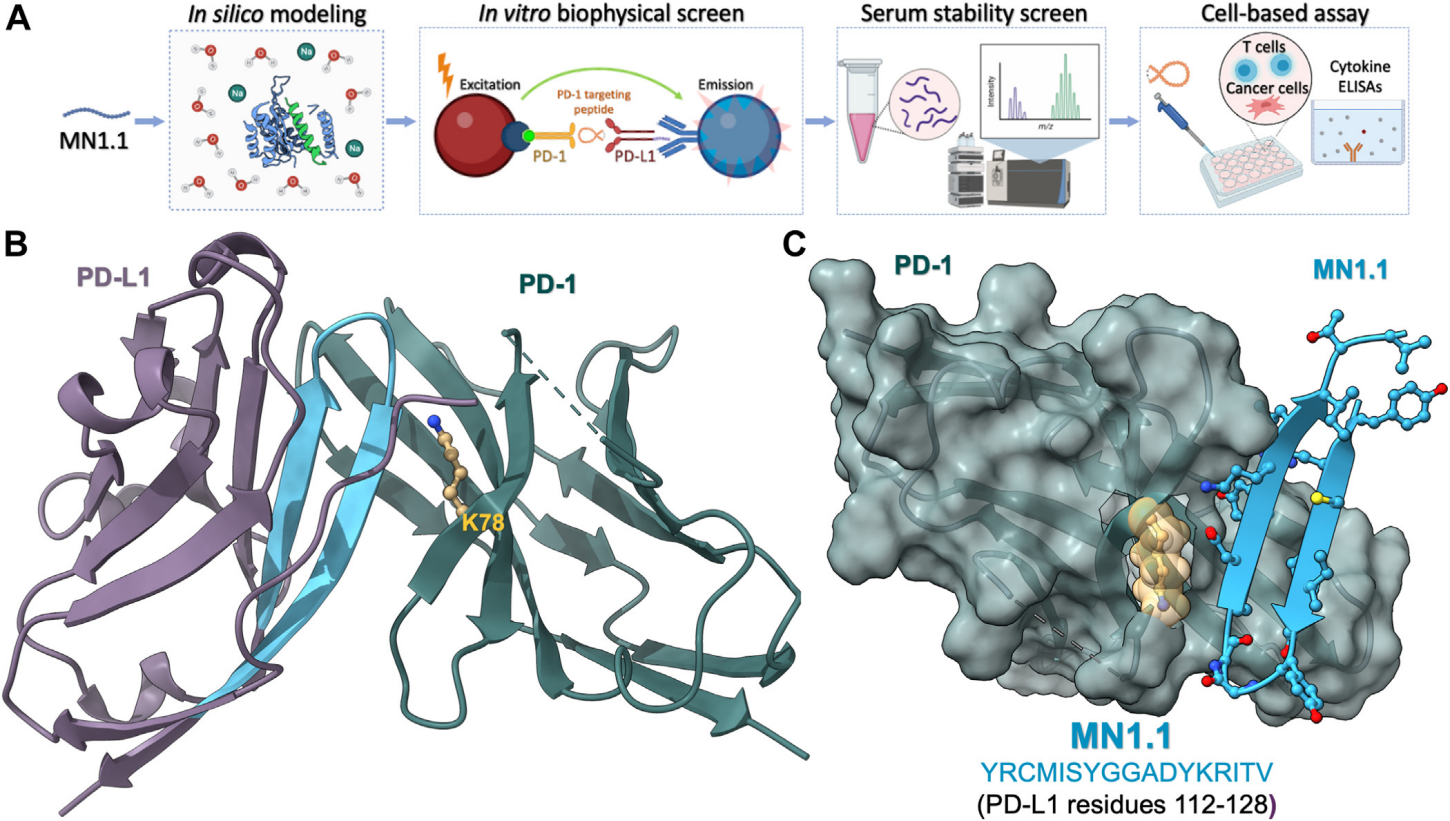
**Experimental overview and design of parent PD-1 targeting peptide, MN1.1.***A*, workflow for MN1.1 optimization and downstream validation. *B*, region of PD-L1 used to design MN1.1 shown in *blue* in the hPD-1/hPD-L1 crystal structure (PDB: 4ZQK); K78 hotspot of PD-1 shown in *gold*. *C*, hPD-1/hPD-L1 structure with only the MN1.1 region of PD-L1. PD-L1, programmed death ligand 1.

In previous work, we characterized the interaction interface of PD-1 and PD-L1 using protein painting, a novel structural biology technique (17, 18). One hotspot of interaction was identified on PD-1 at Lys78, and eight first-generation peptides were designed based on the interface regions identified for PD-1 and PD-L1 (17). The peptide designed from the PD-L1 interface sequence that interacted directly with Lys78 on PD-1 was the most effective at blocking PD-1/PD-L1 complex formation and was chosen as the lead peptide. Our finding was further supported by the published crystal structure of human PD-1/PD-L1, which showed that the region of PD-L1 used to design the lead peptide (PD-L1 residues 112–128) interacted directly with Lys78 of PD-1 (PDB: 4ZQK (19)). Therefore, this lead peptide, MN1.1, was chosen for further optimization to improve affinity for PD-1 and peptide stability. A combination of *in silico* modeling, biophysical, and cell-based assays was used to identify essential binding residues of MN1.1 and investigate the biological activity of optimized candidate peptides (Fig. 1).

### Characterization of MN1.1 binding affinity and design of MN_Null

**Figure 2:**
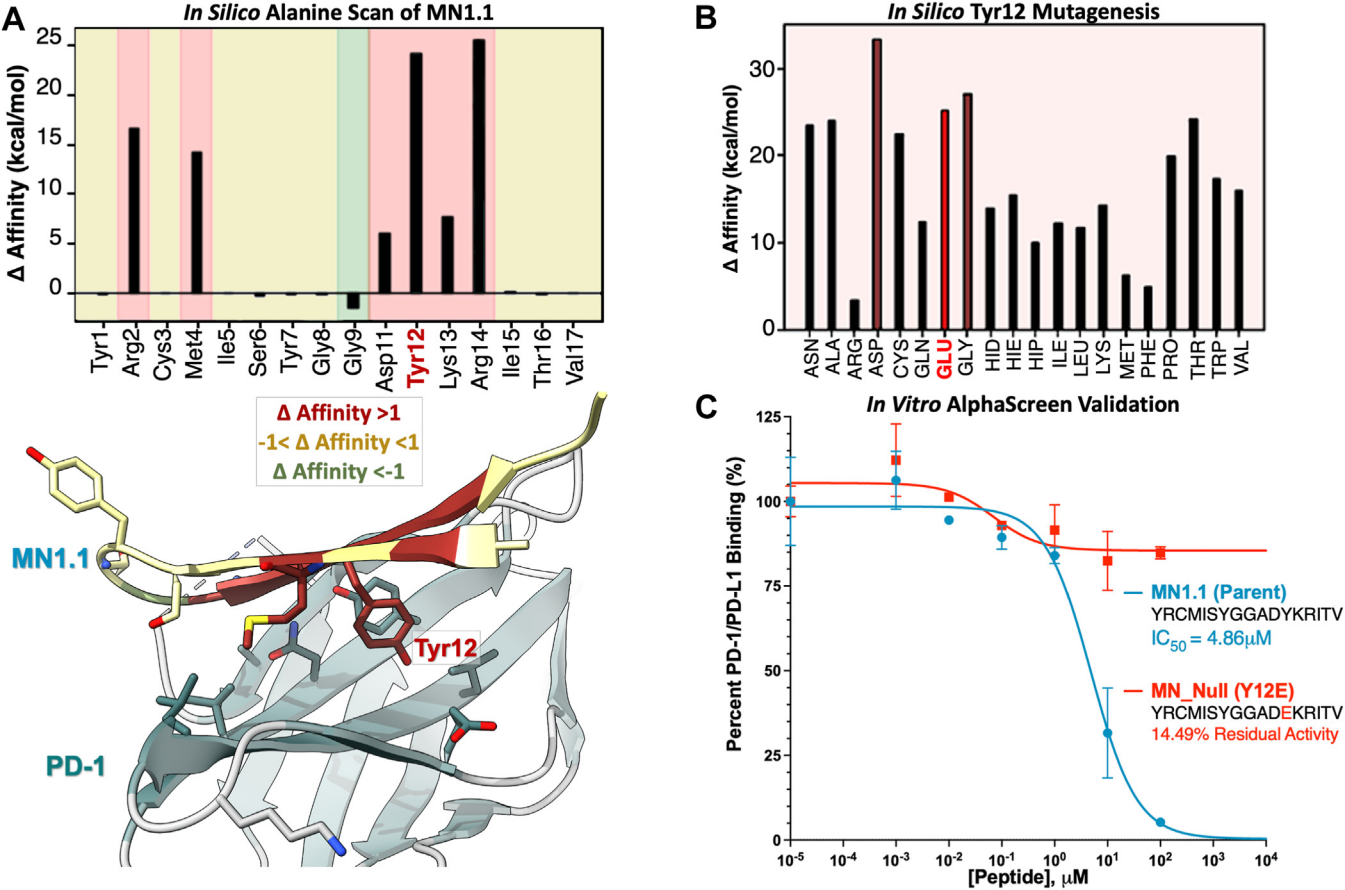
**Prediction of key PD-1 binding residues and *in vitro* validation.***A*, results of MN1.1 computational alanine scan; residues with high affinity for PD-1 are highlighted in *red*, medium affinity in *yellow*, and low affinity in *green* in both the chart and graphical depiction of MN1.1. *B*, results of Tyr12 *in silico* mutagenesis with mutations considered for MN_Null shown in *red*. *C*, *in vitro* validation of MN_Null (Y12E) *via* AlphaScreening.

Key binding residues of MN1.1 were identified by calculating residue-specific contributions to PD-1 binding *via* computational alanine scan. Tyr12 and Arg14 had the greatest positive changes in binding free energy after mutation to alanine, indicating that substitution of these residues was unfavorable for PD-1 binding (Fig. 2*A*). Additionally, molecular mechanics/generalized born surface area (MM/GBSA) calculations were performed to determine the effect of Y12A and R14A mutations on the overall binding free energy of the peptide. The binding free energies were −94.6 kcal/mol for Y12A and −103.4 kcal/mol for R14A. Compared to the −105.8 kcal/mol binding free energy calculated for MN1.1, the Y12A mutation significantly reduced the binding affinity for PD-1. Therefore, the Tyr12 residue was selected for further *in silico* mutagenesis to inform the design of a null peptide for use in downstream experiments. When Tyr12 was computationally mutated to each canonical amino acid, the most unfavorable substitutions were Asp, Glu, and Gly (Fig. 2*B*). Subsequent MM/GBSA calculations demonstrated that the Y12E mutation significantly reduced the binding affinity of the peptide, with a calculated binding free energy of −70.6 kcal/mol. The Y12E mutation was therefore selected to construct the MN_Null peptide. MN1.1 and MN_Null were compared *in vitro via* Amplified Luminescent Proximity Homogeneous Assay (AlphaScreen), a bead-based biophysical screening technique used to measure PD-1/PD-L1 complex formation. AlphaScreen experiments showed that MN1.1 was effective in blocking PD-1/PD-L1 complex formation, whereas the Y12E mutation eliminated the ability of MN_Null to block PD-1/PD-L1 complex formation, as predicted by the modeling data (Fig. 2*C*).

In the hopes of improving the binding affinity of the peptide for PD-1, a linear peptide candidate was generated with mutations informed by our *in silico* data, in addition to cyclized peptides with mutations reported in a recent study of high-affinity PD-L1 mutants (7). However, the linear candidate based on MN1.1 performed worse comparatively when tested *via* AlphaScreen, and the candidates based on a cyclized version of MN1.1 (described in the following section) also displayed weak or no ability to block PD-1/PD-L1 complex formation (Fig. S1, *A* and *B*). This suggested that the sequence of the PD-L1 interface used to design MN1.1 was likely not amenable to amino acid substitution and could possibly be the minimal region of PD-L1 critical for PD-1 binding.

### Stability optimization of MN1.1 and design of MN1.4

Given that many MN1.1 residues appeared crucial for PD-1 binding, modifications to improve peptide stability rather than affinity were pursued, including head-to-tail disulfide bond cyclization and peptide backbone N-methylation, which have been shown to protect against protease degradation (20, 21, 22, 23). A cyclized version of MN1.1, called MN1.2, was created by introducing flanking cysteine residues into the peptide sequence by substituting Tyr1 and Val17 with cysteines. Cys3 was mutated to alanine to prevent erroneous disulfide bond formation at this position. When MN1.1 and MN1.2 were analyzed *via* circular dichroism, the secondary structures of both peptides consisted of approximately half β-loop and half random coil character, indicating that cyclization did not significantly impact peptide structure (Fig. S2*A*).

**Figure 3:**
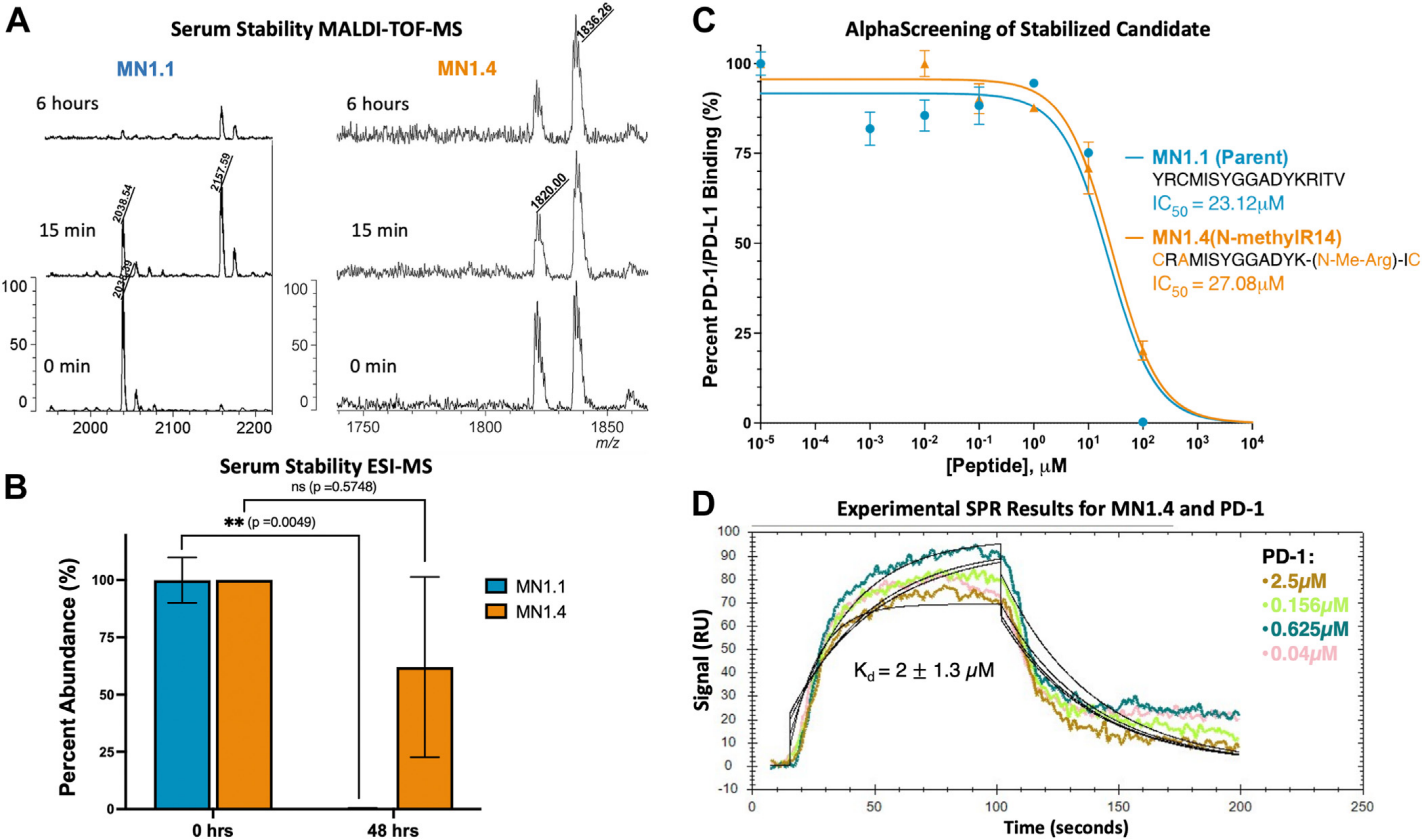
**Serum stability studies and validation of stabilized candidate peptide.***A*, MALDI-TOF-MS results for 6 h time course with MN1.1 (2038 Da) and MN1.4 (1820 Da). *B*, ESI-MS quantification for 48 h time course with MN1.1 and MN1.4; unpaired, two-tailed t-tests were performed for each peptide to compare their respective 0 and 48 h time points. *C*, AlphaScreening of MN1.1 and MN1.4. *D*, representative trial of SPR experiments with PD-1 and MN1.4 (100 μM).

For further stabilization, peptide MN1.4 was created by the addition of an N-methyl group to the MN1.2 peptide backbone (lacking Thr16) to reduce cleavage of the K13-R14 peptide bond by trypsin-like proteases (21, 22, 23). Preliminary serum stability experiments with MN1.1 and MN1.4 revealed that MN1.1 was almost completely degraded after 6 h when analyzed *via* MALDI-TOF-MS (Fig. 3*A*). After 15 min, a new peak for MN1.1 appeared with a mass shift of +119 Da, consistent with cysteinylation of Cys3 of MN1.1 (24). Oxidation of Met4 was observed with MN1.4 (mass shift +16 Da); both forms were present after 6 h without significant degradation. Longer time course experiments analyzed *via* ESI-MS were consistent with the MALDI-TOF-MS results, showing that MN1.1 was completely degraded while MN1.4 was not significantly degraded after 48 h (Fig. 3*B*). MN1.2 was detected at very low levels after 48 h (Fig. S2*B*).

The results demonstrated that the cyclization and N-methylation modifications were successful in improving peptide stability; however, the activity of MN1.4 was compared to MN1.1 *via* AlphaScreen to ensure there were no deleterious effects on activity. The results showed that the modifications introduced to create MN1.4, including N-methylation and mutation or deletion of the Y1, C3, T16, and V17 residues of the parent MN1.1 sequence, did not significantly impact peptide activity (Fig. 3*C*). While oxidation is not a common type of post-translational modification *in vivo* (25), the Met4 residue of MN1.4 was replaced with a structural analog, norleucine, to prevent the oxidation observed *via* MALD-TOF-MS. However, the peptide created with the Met4Nle substitution, MN1.5, was not able to block PD-1/PD-L1 complex formation when tested *via* AlphaScreen, suggesting that the thioether of Met4 could be important for PD-1 binding (Fig. S1*C*). Before testing MN1.4 in a cell-based model, peptide affinity for recombinant PD-1 was measured *via* surface plasmon resonance (Fig. 3*D*). The measured K_d_ of MN1.4 was 2 ± 1.3 μM, a similar order of magnitude as the K_d_ values reported for PD-1/PD-L1 in literature (6, 7, 8, 9). As a stabilized mutant with limited variation from the native sequence, we believed MN1.4 was a compelling probe to investigate the minimal PD-L1 region necessary to trigger PD-1 signaling in a cell-based model.

### Functional characterization of MN1.4 in cell-based assay

**Figure 4:**
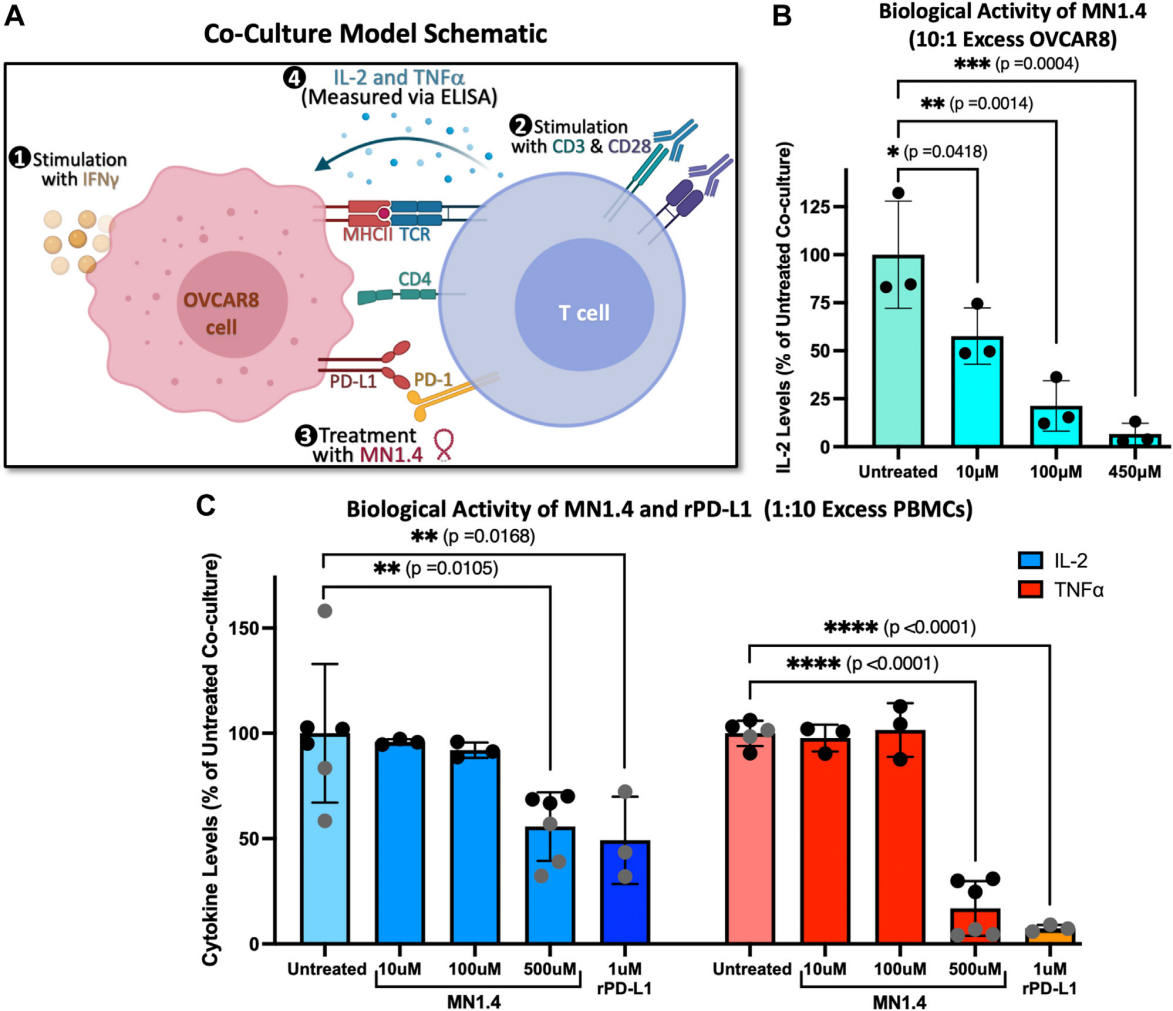
**Functional evaluation of MN1.4 in a co-culture model.***A*, schematic overview of co-culture model. *B*, IL-2 PCR-ELISA results using a 10:1 OVCAR8:PBMC co-culture ratio. IL-2 levels normalized to untreated co-culture control. Ordinary one-way ANOVA was performed with Dunnett’s multiple comparison test, with a single pooled variance to compare the treatment groups to the untreated control (run in triplicate). *C*, IL-2 and TNFα ELISA results for MN1.4 and recombinant PD-L1 using a 1:10 OVCAR8:PBMC ratio. Cytokine levels normalized to untreated co-culture control. Ordinary one-way ANOVAs were performed with Dunnett’s multiple comparison test, with a single pooled variance to compare the IL-2 and TNFα treatment groups to their respective untreated controls. Treatment groups run in triplicate for each biological replicate (differentiated by *black vs. gray* data points). PD-L1, programmed death ligand 1.

A co-culture model with OVCAR8 cells and human peripheral blood mononuclear cells (PBMCs) was designed to examine the immunomodulatory effects of MN1.4 treatment in the context of ovarian cancer. For this model, PBMCs and cancer cells were both stimulated prior to co-culture to promote expression of PD-1 and PD-L1, respectively (Fig. S3). T cell activation was measured by quantifying levels of secreted cytokines *via* ELISA after 24 h treatment with MN1.4. Initial optimization of the model included testing different co-culture ratios of OVCAR8 cells to PBMCs to determine if excess cancer cells or excess T cells would provide the best dynamic range for measuring differences in cytokine levels between treatment groups (Fig. S4*A*). Further optimization of the co-culture protocol and preliminary testing of MN1.4 was conducted using a 10:1 co-culture ratio of OVCAR8:PBMCs and was analyzed using an IL-2 PCR-ELISA hybrid kit (Fig. 4*B*).

The results of the preliminary trial suggested MN1.4 enhanced the suppression of IL-2 secretion by T cells in a dose-dependent manner. However, IL-2 levels detected in this trial were very low (<6 pg/ml), likely due to low PBMC concentrations given the 10:1 ratio of excess OVCAR8 cells used in this experiment. Therefore, experiments were repeated with a 1:10 ratio of OVCAR8:PBMCs to increase the dynamic range of the assay and improve statistical confidence. In addition, TNFα, a classical marker of T cell activation, was measured *via* traditional ELISA to ensure the effects of MN1.4 were monitored using multiple outputs. Consistent with the results from the 10:1 co-culture, a similar pattern of T cell suppression was observed when IL-2 and TNFα levels were measured in the 1:10 co-cultures *via* ELISA (Fig. 4*C*). Furthermore, TNFα experiments with MN_Null showed less immunosuppressive activity as compared to MN1.4 (Fig. S4*B*). Therefore, MN1.4, designed based on the interface sequence of PD-L1, appeared to function as a PD-1 agonist. Additionally, when the biological activity of MN1.4 was compared to full-length, recombinant PD-L1 (rPD-L1) using the co-culture model, results showed that MN1.4 and rPD-L1 significantly decreased IL-2 and TNFα levels in a similar manner compared to the untreated co-culture control (Fig. 4*C*). Therefore, MN1.4 appeared to contain the functionally relevant sequence of PD-L1 required to trigger PD-1 signaling and suppress T cell responses.

## Discussion

While recent studies have examined PD-1 agonist antibodies or fusion proteins mimicking PD-L1, few have focused on functional characterization of the native PD-L1 interface sequence to determine the region essential for triggering PD-1 signaling (26, 27, 28, 29). Previously reported peptides mimicking the PD-L1 interface were designed as PD-1 inhibitors (13, 15, 27), and earlier studies either introduced significant modifications to the native PD-L1 sequence or did not include biological characterization experiments, such as testing in a cell-based model. In this work, we identified and characterized the functional region of PD-L1 required for binding and initiation of PD-1 signaling. To our knowledge, this is the first study to compare the biological function of the PD-L1 interface region to the function of native, full-length PD-L1. An optimized peptide, MN1.4, was designed with little variation from the native PD-L1 interface sequence, with the exception of substitutions near the terminal ends required for cyclization (Tyr1, Cys3, Thr16, Val17). The results of computational alanine scanning and *in vitro* experiments suggested that mutations were not tolerated between Met4-Arg14, including mutations to alanine or simple changes to sidechain chemistry, indicating that the corresponding region of PD-L1 was crucial for PD-1 binding. MN1.4 underwent functional characterization in a cell-based model and displayed similar immunomodulatory activity as full-length PD-L1. Therefore, MN1.4 functions as a PD-1 agonist and likely contains the biologically active sequence of PD-L1 required to trigger PD-1 signaling and suppress T cell responses.

Future directions include comparing the affinity of peptides built from the MN1.4 scaffold to determine if an affinity threshold exists for transitioning a PD-1 agonist to an antagonist. While this hypothesis was not fully explored in this work, it is possible that ligand affinity for PD-1 could be the delineating factor between agonists and antagonists, in which molecules with affinities in the low micromolar range, similar to MN1.4 and native PD-L1, could function as agonists, whereas those with higher affinities could function as antagonists. The lowest affinity reported for a protein antagonist of PD-1 is 750 nM compared to 17 μM of native PD-L1 (7); suggesting that a 20-fold improvement in affinity over the native protein is sufficient to transition to antagonism. Given the peptide sequence of MN1.1 was not amenable to amino acid substitution, modulating structural rigidity could be explored as a means of improving affinity. Ideally, further optimization efforts will improve the potency of MN1.4 while maintaining agonism, and the resulting optimized peptides will undergo testing in mouse models of diseases caused by deficient PD-1 signaling, such as C57BL/6 *PD-1^−/−^* mice that have been used to study PD-1 deficiency in the context of lupus-like disease and type 1 diabetes (3, 30). While the PD-1/PD-L1 pathway is not the only pathway implicated in autoimmunity, peptides optimized from the MN1.4 scaffold could be used to treat several common diseases shown to have deficient PD-1/PD-L1 signaling, such as diabetes and arthritis, in addition to autoimmune diseases, such as those caused by conventional mAb-based immunotherapies (31, 32). Such an optimized compound could be a first-in-class immunosuppressant without some of the associated downsides of steroids and other standards of care.

## Experimental procedures

### Computational alanine scanning

Computational alanine scanning was performed using the Schrӧdinger Bioluminate Suite Residue Scanning feature in Schrӧdinger Maestro version 2020.2. The crystal structure of hPD-1/PD-L1 (PDB: 4ZQK (19)) was modified to include full-length PD-1 and only the PD-L1 residues corresponding to MN1.1 (residues 112–128). Changes in the binding free energy of each residue in the MN1.1 region of PD-L1 were calculated after mutation to alanine, and the effect of select mutations on the binding free energy of the overall peptide was determined *via* MM/GBSA calculations performed in Schrӧdinger Maestro. Residues of interest underwent further *in silico* mutagenesis to each canonical amino acid to predict mutations that would either reduce or enhance binding to PD-1. MM/GBSA calculations were performed for selected mutations to calculate binding free energy changes at the peptide level. All parameter, starting coordinate PDBs, and script files are provided on a public Open Science Framework page (https://osf.io/82n73/).

### AlphaScreening experiments

MN1.1, MN_Null, MN2.1, MN3.2, MN4.2, MN1.4, and MN1.5 were synthesized by Peptide 2.0 Inc. and received as lyophilized trifluoroacetic acid (TFA) salts (sequences provided in Table S1). All peptides were quality-controlled by Peptide 2.0 *via* high-performance liquid chromatography-mass spectrometry to determine purity and verify peptide sequence. Peptides were screened using the PerkinElmer PD-1/PD-L1 (human) AlphaLISA Binding Kit (Cat. No. AL356HV) according to the manufacturer’s instructions and luminescence was measured using a Tecan Spark multimode plate reader with the following modifications: luminescence readings for MN1.1 head-to-head comparisons with MN_Null and MN2.1 were measured at room temperature, and all other trials were measured at 4 °C. Nonlinear regression analysis was conducted using GraphPad Prism version 10; samples were run in triplicate with ROUT outlier analysis.

### Serum stability experiments

Peptide stocks for MN1.1, MN1.2, and MN1.4 (Peptide 2.0 Inc) were prepared at a concentration of 10 μg/μl in DMSO. Human serum was diluted 1:4 in Gibco RPMI 1640 media (ThermoFisher Scientific, Cat. No. 11–875–093) and incubated at 37 °C for 15 min before peptide stock was added at final concentration of 10 μg/ml. Time points were taken at room temperature and mixed with 200 μl of 3% TFA before a 15 min incubation on ice. Samples were centrifuged at 16,000*g* for 2.5 min and the supernatant was prepared for C18 desalting by adding 100 μl of 20% acetonitrile, 2% TFA before storage at −20 °C overnight. Samples were processed through Pierce C18 spin columns (ThermoFisher Scientific, Cat. No. 89870) according to the manufacturer’s instructions. Desalted samples were dried under nitrogen and stored at −20 °C until mass spectrometry analysis.

For MALDI-TOF-MS analysis, samples were reconstituted in 200 μl of 0.1% formic acid. Samples were diluted 1:10 in a MALDI matrix containing a 50 fmol/μl solution of Angiotensin I as a control, spotted onto the plate at a volume of 1 μl, and run on an Axima Performance MALDI TOF/TOF mass spectrometer (Shimadzu Scientific Instruments). Data was visualized with Axima Launchpad 2.8 software (Shimadzu Scientific Instruments). For ESI-MS analysis, samples were run on an Orbitrap Exploris 480 (ThermoFisher Scientific). Samples were spiked with 25 fmol Angiotensin I as an internal control. Peptides were separated using reversed-phase PepMap RSLC 75 μm i.d. x 15 cm long with 2 μm particle size, C18 resin LC column (ThermoFisher Scientific, Cat. No. 164534). Mobile phase A consisted of 0.1% aqueous formic acid and mobile phase B consisted of 0.1% formic acid in 80% acetonitrile. After sample injection (2 μl), peptides were eluted using a linear gradient from 5 to 50% B over 30 min, ramping to 100% B for an additional 2 min. The flow rate was set at 300 nl/min. The mass spectrometer was operated in a data-dependent mode. One full MS scan (60,000 resolving power) from 300 to 1800 m/z using quadrupole isolation was followed by MS/MS scans to select and fragment the most abundant molecular ions by higher energy collisional dissociation using a normalized collision energy of 27%. ThermoFisher’s Xcalibur 4.0 Data System was used for peptide quantification *via* peak area (normalized based on Angiotensin I abundance).

### Surface plasmon resonance

Biotinylated (N-terminus modified) MN1.4 (Peptide 2.0 Inc) was immobilized at a concentration of 100 μM on Nicoya streptavidin-conjugated gold sensor chips (Cat. No. SEN-AU-100–10) and inserted into the flow chamber of a benchtop Nicoya OpenSPR 2-channel device. Dilutions of recombinant human PD-1 (2.5 μM, 625 nM, 156 nM, 40 nM) from R&D Systems (Cat. No. 8986-PD-100) were prepared in sterile PBS and allowed to flow over the chip at a flow rate of 50 μl/min. Traces were evaluated using the TraceDrawer software. The presented K_d_ value is the average of two independent trials.

### Cell culture conditions

OVCAR8 cells, generously provided by the Gottesman Lab (National Institutes of Health), were cultured in Gibco RPMI 1640 with 10% Gibco fetal bovine serum (ThermoFisher Scientific, Cat. No. 10437028), 1% penicillin/streptomycin (10,000 U/ml pen, 10 mg/ml strep) from MilliporeSigma (Cat. No. P4333). Cells were cultured at 37 °C and 5% CO_2_. The passage number was kept below three for co-culture assay. Human peripheral blood mononuclear cells were purchased frozen from STEMCELL Technologies (Cat. No. 70025.2) from two female donors. PBMCs cultured in the same media and conditions as cancer cell lines.

### Co-culture conditions

For co-culture experiments with excess OVCAR8 ratios, OVCAR8 cells were seeded at 2 × 10^5^ cells/well on a 6-well plate and allowed to adhere overnight. The next day, cells were stimulated with 100 ng/ml IFNγ (R&D Systems, Cat. No. 285-IF-100), and two vials of human PBMCs were thawed according to manufacturer’s instructions. On day 3, concentrations of PBMCs and OVCAR8 cells were measured using QuadCount Automated Cell Counter (Accuris Instruments). The concentration of PBMCs required for a 10:1 OVCAR8:PBMC ratio was calculated from the OVCAR8 concentration. PBMCs were seeded on a separate 6-well plate (1 ml/well) and treated with MN1.4 (450 μM, 100 μM, or 10 μM) from Lifetein for 30 min prior to co-culture. After 30 min, media was removed from OVCAR8 wells and replaced with 1 ml of treated PBMCs. PBMCs were stimulated 30 min after co-culture with 5 μg/ml of anti-CD3 (Cat. No. 555329) and 5 μg/ml of anti-CD28 (Cat. No. 567117) from BD Biosciences. After 24 h, the supernatants were collected from each well, and centrifuged at 10,000*g* for 5 min.

For co-culture experiments with excess PBMCs ratios, OVCAR8 cells were seeded at 2 × 10^4^ cells/well on a 24-well plate and allowed to adhere overnight. The next day, cells were stimulated with 100 ng/ml IFNγ and two vials of human PBMCs were thawed. On day 3, concentrations of PBMCs and OVCAR8 cells were measured. The concentration of PBMCs required for a 1:10 OVCAR8:PBMC ratio was calculated from the OVCAR8 concentration. PBMCs were plated on a separate 24-well plate (1 ml/well) and stimulated with 5 μg/ml of anti-CD3 and 5 μg/ml of anti-CD28 for 30 min before drug treatment. PBMCs were treated with MN1.4 (500 μM, 100 μM, or 10 μM) from Peptide 2.0 or 1 μM recombinant human PD-L1 (R&D systems, Cat. No. 156-B7-100) for 30 min prior to co-culture. After 30 min, media was removed from OVCAR8 wells and replaced with 1 ml of treated PBMCs. After 24 h, the supernatants were collected from each well, and centrifuged at 10,000*g* for 5 min.

### Co-culture ELISAs and data analysis

For co-culture experiments analyzed *via* IL-2 PCR-ELISA, supernatants were processed using the IL-2 Human ProQuantum Immunoassay Kit (ThermoFisher Scientific, Cat. No. A35603) according to manufacturer’s instructions with the following modifications. The low-volume protocol was followed for samples from excess OVCAR8 co-culture experiments and antibody control experiments. Samples were diluted 1:10 in assay buffer, and were allowed to incubate with the IL-2 antibody-oligonucleotide conjugate overnight at 4 °C. The high-volume protocol was followed for samples from excess PBMC co-culture experiments. Samples were diluted 1:5, and were allowed to incubate for 1 h at room temperature. PCR was run according to the manufacturer’s instructions using the QuantStudio seven Pro Real-Time PCR System equipped with a 96-well, 0.2 ml block from ThermoFisher Scientific. TNFα levels were also assessed in excess PBMC co-culture samples using TNFα ELISA MAX Deluxe Set from BioLegend (Cat. No. 430204) according to manufacturer’s instructions. Absorbance was measured using a Tecan Spark multimode plate reader. GraphPad Prism version 10 was used to perform nonlinear regression analyses to generate standard curves and interpolate cytokine levels. Ordinary one-way ANOVAs were performed with Dunnett’s multiple comparison test, with a single pooled variance to compare each treatment group to the untreated co-culture control. ROUT outlier analysis was performed to identify and remove any outliers from the analyses. Values for each treatment condition were normalized to the untreated control.

### Circular dichroism

For CD experiments, lyophilized stocks of MN1.1 and MN1.2 (Peptide 2.0 Inc) were reconstituted in 10 mM sodium phosphate buffer (pH 7) to a final concentration of 75 μM. Baselines were run for each sample with 10 mM sodium phosphate buffer (pH 7). All spectra were collected with a J-1500 Circular Dichroism Spectrophotometer (Jasco) equipped with a Jasco PTC-517 temperature controller using 0.1 cm pathlength quartz cuvettes (Jasco, Cat. No. 0556). Spectra were collected at 20 °C in the 190 to 300 nm range at a 50 nm/min scanning speed, 1 nm bandwidth, and 1 nm data pitch over an average of three scans. CD spectra were plotted using GraphPad version 9. Baseline-corrected data were uploaded to DichroWeb (http://dichroweb.cryst.bbk.ac.uk/html/home.shtml) (33) and deconvoluted using the CDSSTR algorithm (34) and reference set SMP180t (35) to report estimated secondary structure percentages.

## Data availability

All data are provided in the manuscript with the following exceptions. Peptide sequences are provided in Supporting Information in Table S1, along with AlphaScreening data for mutated candidate peptides in Fig. S1. Structure comparison of MN1.1 and MN1.2 are provided in Supporting Information in Fig. S2. Co-culture control experiments are provided in Supporting Information in Figs. S3 and S4.

## Supporting information

This article contains supporting information (7).

## Conflict of interest

R. C., A. H., A. B., A. S., M. P., L. L., and A. L. are inventors on patents related to protein painting and disclosed structures. Monet Pharmaceuticals licensed the rights of patents related to protein painting that are owned by George Mason University. A. H., L. L., and A. L. own shares of Monet Pharmaceuticals.

The remaining authors declare that the research was conducted in the absence of any commercial or financial relationships that could be construed as a potential conflict of interest.
