# Supplementary material for: Identification of the functional PD-L1 interface region responsible for PD-1 binding and initiation of PD-1 signaling
Source: J Biol Chem. 2023 Oct 17;299(12):105353. doi: 10.1016/j.jbc.2023.105353 (PMC10663846; doi:10.1016/j.jbc.2023.105353)
Supplement: Supporting information [file mmc1.docx]

***Supporting Information***

**Supplementary Table 1.** Peptide sequences (in order of appearance in manuscript).

| Peptide | Sequence | Optimization Principle |
| --- | --- | --- |
| MN 1.1 | Ac-YRCMISYGGADYKRITV-NH_2_ | None (Parent) |
| MN_Null | Ac-YRCMISYGGADEKRITV-NH_2_ | Abolish affinity |
| MN 1.2 | Ac-CRAMISYGGADYKRITC-NH_2_ | Reduce cysteinylation, protease degradation |
| MN 1.4 | NH_2_-CRAMISYGGADYK-(N-Me-Arg)-IC-COOH | Reduce cysteinylation, protease degradation |
| MN 1.5 | NH_2_-CRA-Nle-ISYGGADYK-(N-Me-Arg)-IC-COOH | Reduce oxidation, cysteinylation, protease degradation |
| MN 2.1 | Ac-YRCMISNGGADYKRITV-NH_2_ | Improve affinity |
| MN 4.2 | NH_2_-CRALIAYKGADYKRIC-COOH | Improve affinity |
| MN 3.2 | NH_2_-CRAMISYKGADYKRIC-COOH | Improve affinity |


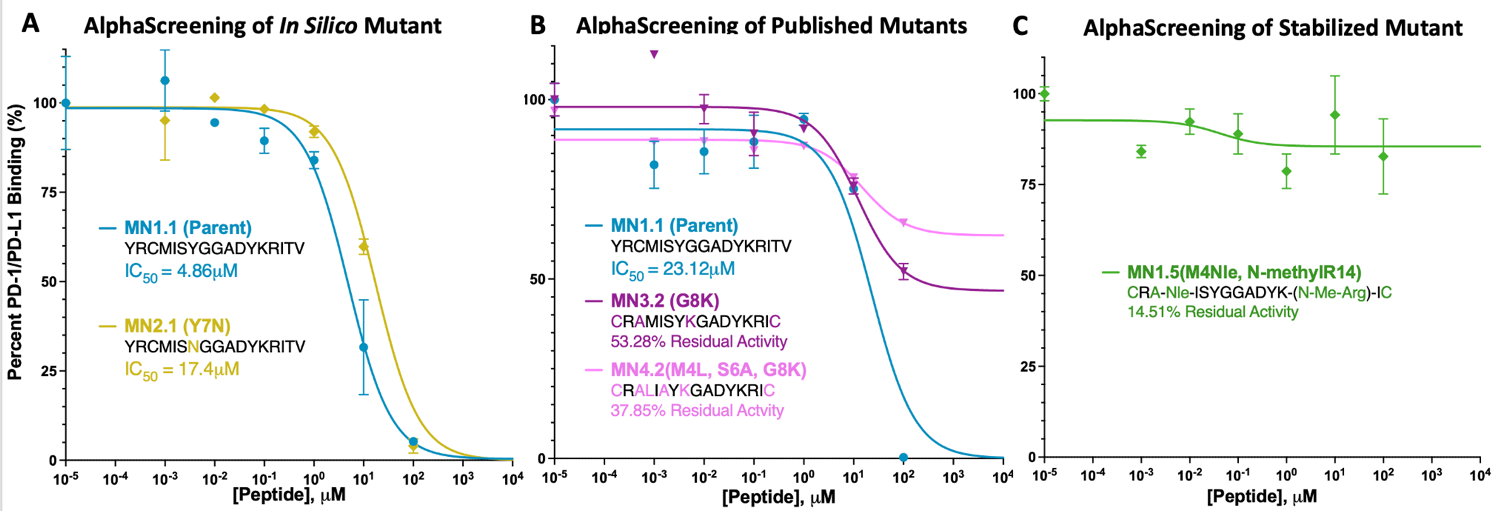


**Supplementary Figure 1. AlphaScreening results for candidate peptides indicate limited toleration of amino acid substitution in MN1.1.** (A) MN1.1 compared to MN2.1, a peptide with a Y7N mutation predicted to improve affinity via *in silico* modeling. (B) MN1.1 compared to MN4.2 and MN3.2, cyclized peptides with mutations reported for published high-affinity PD-L1 mutants^7^. (C) MN1.5, a version of stabilized peptide MN1.4 with a Met4Nle substitution to reduce Met4 oxidation.


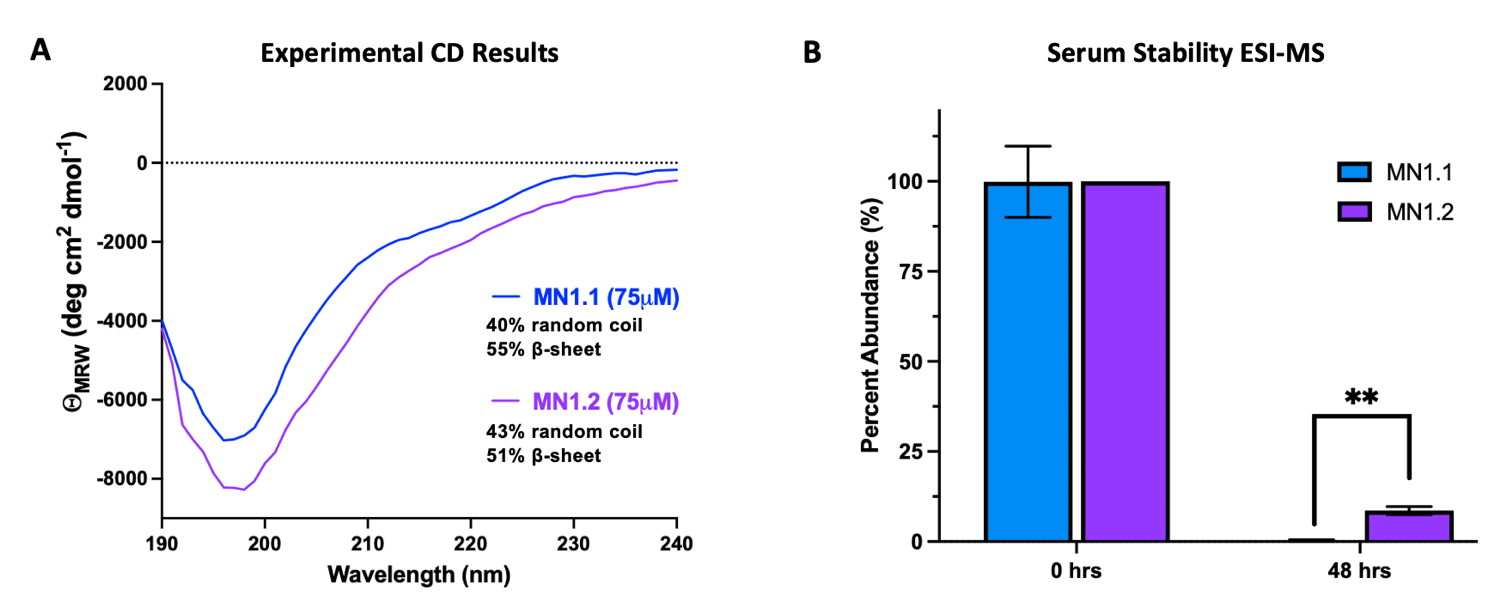


**Supplementary Figure 2. Circular dichroism and serum stability experiments indicate that disulfide bond cyclization of MN1.1 does not significantly impact peptide structure but improves serum stability.** (A) Circular dichroism characterization of MN1.1 and MN1.2, a cyclized version with flanking Cys residues and a C3A mutation. (B) ESI-MS quantification for 48-hour serum stability time course with MN1.1 and MN1.2; an unpaired, two-tailed t-test was used to compare the 48-hour time points.


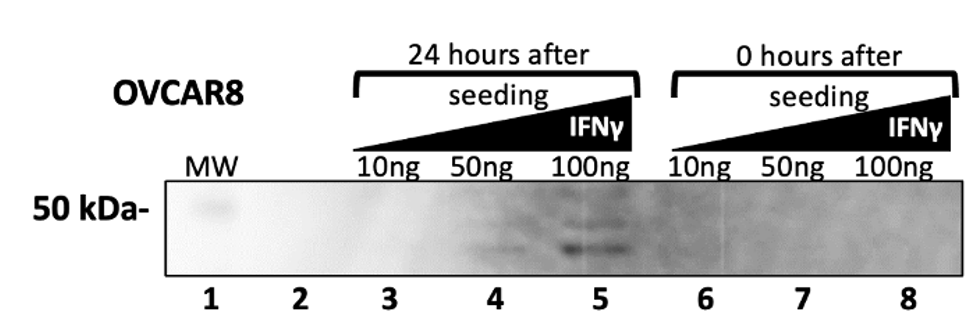


**Supplementary Figure 3**. **Western blot for PD-L1 in OVCAR8 cells after IFNγ treatment.** OVCAR8 cells were treated with increasing concentrations of IFNγ either immediately upon seeding or 24 hours after seeding. IFNγ treatment 24 hours after seeding was more effective for stimulating PD-L1 expression.


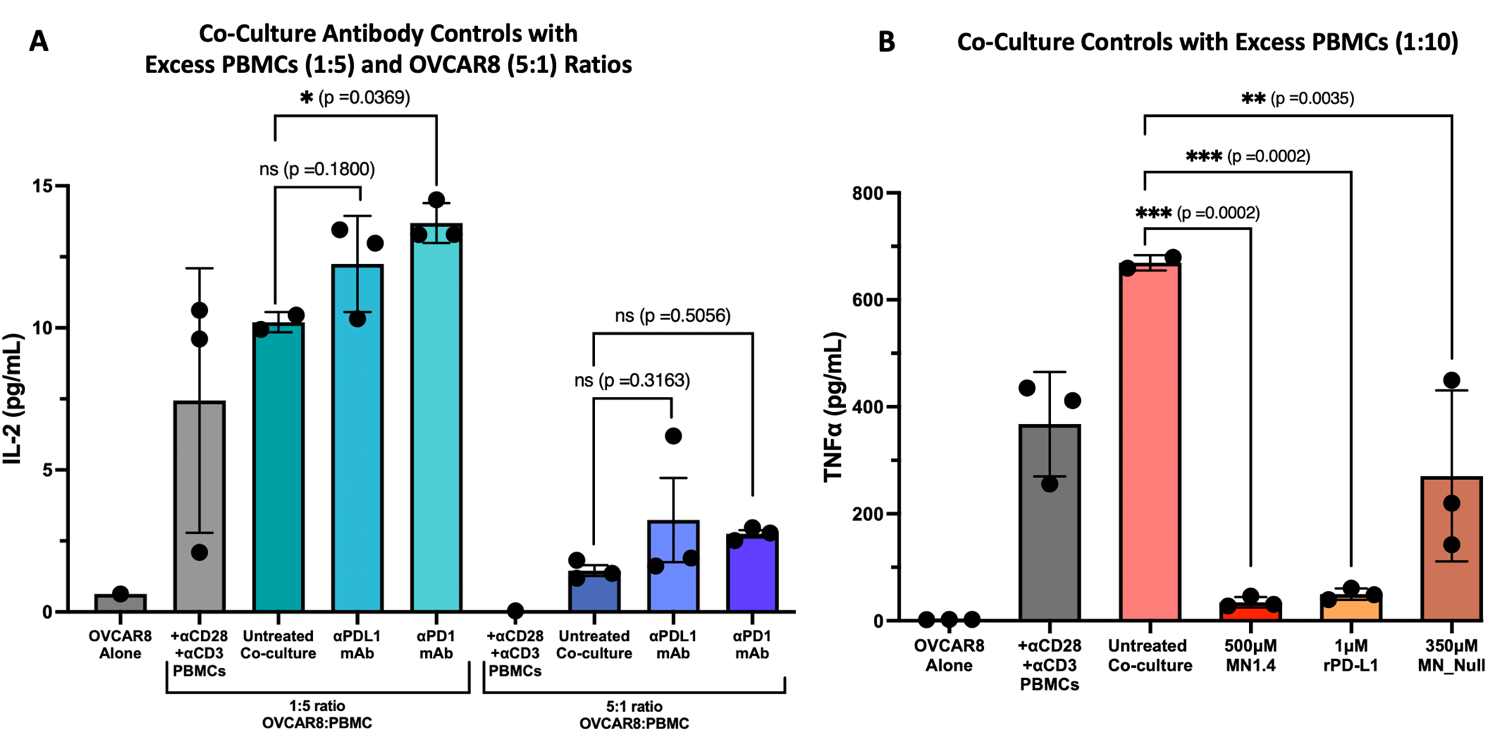
**Supplementary Figure 4**. **Co-culture ratio optimization results and co-culture control experiments.** (A) IL-2 PCR-ELISA results using a 1:5 ratio of OVCAR8:PBMC (cell densities on day of co-culture were 3.2x10^5^ OVCAR8/mL and 1.6x10^6^ PBMCs/mL) and a 5:1 ratio of OVCAR8:PBMC (cell densities on day of co-culture were 3.2x10^5^ OVCAR8/mL and 6.4x10^4^ PBMCs/mL) with PBMCs from Donor 2. Cells were treated with 200 nM of Pembrolizumab (Biovision, Milpitas, CA, USA, Cat. No. A1306-100) or Atezolizumab (Biovision, Cat. No. A1305-100) for 24 hours. (B) TNFα ELISA results using an OVCAR8:PBMC ratio of 1:10 and PBMCs from Donor 1 (cell densities on day of co-culture were 1.45x10^5^ OVCAR8/mL and 1.45x10^6^ PBMCs/mL). Cells treated with drug for 24 hours. Ordinary one-way ANOVA was performed with Dunnett’s multiple comparison test, with a single pooled variance to compare the 1:5 and 5:1 treatment groups to their respective untreated controls.
